# Preparation of bilayer tissue-engineered polyurethane/poly-L-lactic acid nerve conduits and their in vitro characterization for use in peripheral nerve regeneration

**DOI:** 10.1186/s13036-024-00412-9

**Published:** 2024-02-22

**Authors:** Mehran Nabipour, Amir Mellati, Mozhgan Abasi, Somayeh Ebrahimi Barough, Ayoob Karimizade, Parnian Banikarimi, Elham Hasanzadeh

**Affiliations:** 1 Department of Tissue Engineering & Regenerative Medicine, School of Advanced Technologies in Medicine, Mazandaran University of Medical Sciences, Sari, Iran; 2 Student Research Committee, School of Advanced Technologies in Medicine, Mazandaran University of Medical Sciences, Sari, Iran; 3 Molecular and Cell Biology Research Center, Faculty of Medicine, Mazandaran University of Medical Sciences, Sari, Iran; 4 Immunogenetics Research Center, Mazandaran University of Medical Sciences, Sari, Iran; 5 Department of Tissue Engineering, School of Advanced Technologies in Medicine, Tehran University of Medical Sciences, Tehran, Iran

**Keywords:** Tissue engineering, Polyurethane, Carbon nanotube, Nanofibrous scaffolds, Poly-L-lactic acid, Nerve conduit, Sciatic nerve injury, Human endometrial stem cells, Neural differentiation

## Abstract

**Background:**

Due to loss of peripheral nerve structure and/or function resulting from trauma, accidents, and other causes, peripheral nerve injuries continue to be a major clinical problem. These injuries can cause partial or total loss of sensory, motor, and autonomic capabilities as well as neuropathic pain. PNI affects between 13 and 23 out of every 100,000 people annually in developed countries. Regeneration of damaged nerves and restoration of function after peripheral nerve injury remain significant therapeutic challenges. Although autologous nerve graft transplantation is a viable therapy option in several clinical conditions, donor site morbidity and a lack of donor tissue often hinder full functional recovery. Biomimetic conduits used in tissue engineering to encourage and direct peripheral nerve regeneration by providing a suitable microenvironment for nerve ingrowth are only one example of the cutting-edge methods made possible by this field. Many innate extracellular matrix (ECM) structures of different tissues can be successfully mimicked by nanofibrous scaffolds. Nanofibrous scaffolds can closely mimic the surface structure and morphology of native ECMs of many tissues.

**Methods:**

In this study, we have produced bilayer nanofibrous nerve conduit based on poly-lactic acid/polyurethane/multiwall carbon nanotube (PLA/PU/MWCNT), for application as composite scaffolds for static nerve tissue engineering. The contact angle was indicated to show the hydrophilicity properties of electrospun nanofibers. The SEM images were analyzed to determine the fiber**’**s diameters, scaffold morphology, and endometrial stem cell adhesion. Moreover, MTT assay and DAPI staining were used to show the viability and proliferation of endometrial stem cells.

**Results:**

The constructed bilayer PLA/PU/MWCNT scaffolds demonstrated the capacity to support cell attachment, and the vitality of samples was assessed using SEM, MTT assay, and DAPI staining technique.

**Conclusions:**

According to an in vitro study, electrospun bilayer PLA/PU/MWCNT scaffolds can encourage the adhesion and proliferation of human endometrial stem cells (hEnSCs) and create the ideal environment for increasing cell survival.

## Background

One of the widespread clinical issues that impair the patient’s quality of life is peripheral nerve injury (PNI). Peripheral nerves have the ability to regenerate after damage, but the whole process might not be enough to provide a full functional repair. The age of the patient, the location and kind of lesion, the injured nerve trunk, nerve gap distance, the surgical repair method, and the amount of time between the injury and therapy are some of the variables affecting functional recovery [1, 2]. According to Reyes et al., neurological recovery is generally regarded as moderate in humans for nerve gaps smaller than 2 cm, but minimal to non-existent for gaps larger than 4 cm. However, protecting pain sensitivity and motor strength in partially denervated muscles are typically the only benefits of collateral reinnervation by undamaged axons, which is constrained by spatial and temporal limitations, particularly for big sensory and motor axons [2–4].

In order for an injured or degenerated nerve to regenerate, certain conditions must be met. Firstly, the distance between the proximal and distal ends of the injured nerve should not exceed the critical nerve gap, which is greater than 1.5 cm for rats, greater than 3 cm for rabbits, and greater than 4 cm for pigs/humans. Secondly, the axons must be surrounded by neurilemma and endoneurium. Thirdly, the cell body must have an intact nucleus. Lastly, the two separated ends of the nerve should remain in the same plane of injury [5–7].

The existing treatment options for PNI can be categorized into 2 main approaches, surgical and non-surgical. Electrical and magnetic stimulators, laser phototherapy, and nerve growth factors are examples of non-surgical treatment approaches [8]. Methods like neurorrhaphy, grafting (allografts and autografts nerves), and tissue engineering grafts are examples of surgical treatment methods [9]. Recently, tissue engineering has been proposed as a novel and different therapeutic strategy for peripheral nerve grafting or tissue transplantation. A biomaterial scaffold is utilized as the ECM for cell transplantation and regeneration of damaged central and peripheral nerve systems in tissue engineering approaches [10, 11].

A biologically interacting microenvironment, a biomechanically robust structure resembling native nerve tissue, and topographically biomimetic architecture are all requirements for appropriate tissue scaffolds. Recently, several artificial neural guidance conduits have been used to get around the problems associated with nerve autografts. Therefore, creating bioactive scaffolds for the purpose of fabricating nerve conduction channels is a promising alternative to autograft [12, 13].

Different artificial nerve guide implants have undergone testing as substitutes for autologous nerve grafts. A variety of materials, including extracellular matrix components and synthetic polymers, have been utilized in animal experiments that include nerve tubulation. Additionally, additives such as recombinant proteins or implanted cells that produce growth factors have also been employed [14]. Initial experimentation with humans involved the utilization of basic silicone tubes [14, 15].

Current research on synthetic nerve guides has only included several different materials in published human investigations [16–18]. For example, silicone and polytetrafluoroethylene (PTFE) are two non-resorbable inert polymers, while Poly (glycolic acid) (PGA) and polylactide-caprolactone (PLCL) are two resorbable synthetic polymers. The initial authorization of nerve guidance occurred in 1999. The authorized goods consist of hollow tubes fabricated from resorbable materials such as PLCL, PGA, collagen, or a non-resorbable hydrogel based on polyvinyl alcohol. All approved implants are clearly constrained in their length and serve as a substitute for autologous neural implants in cases of short nerve deficits. Several studies suggest that extended faults necessitate sophisticated implants that are currently being researched [14].

Biomaterial selection is one of the most important parameters in scaffold designs for tissue engineering approaches. Different groups of biomaterials can be applied to enhance nerve regeneration from natural to synthetic [19]. Collagen, gelatin, laminin, and chitosan are some of the common natural biomaterials that are used in the case of regeneration. In addition, due to some of the shortages of natural biomaterials, the application of synthetic biomaterials is prevalent in nerve regeneration. Poly (glycolic acid) (PGA), poly (lactide acid) (PLA), poly (caprolactone) (PCL), poly (lactic-co-glycolic acid) (PLGA), and polyurethane (PU) are some of the examples in this category [19, 20].

Synthetic polyester, PLA, has proven to be very useful in tissue engineering. An aliphatic polyester, this polymer has mostly been used in biomedical applications. It is an organic substance with predictable kinetics of degradation, minimal allergenic potential, low toxicity, and excellent biocompatibility [21]. PLA can be created either through ring-opening polymerization or polycondensation, and it hydrolytically breaks down into the metabolic byproduct lactic acid, making it appropriate for use in medicinal applications. Moreover, PLA nanofibers have demonstrated a variety of applications in the fields of neural tissue engineering, drug delivery, and regenerative medicine [22]. To design and construct scaffolds with supporting properties for Schwann cells, allowing elongation of axons, and promoting vascular growth, PLA can be one of the appropriate choices [23].

By considering the unique chemical, mechanical, and thermal characteristics of PU, this synthetic polymer has been applied for fabricating tissue engineering scaffolds in many researches. It was shown that electrospun PU fibers are excellent candidates for soft tissue engineering, such as CNS [24, 25]. High surface area, biomimicry of natural extracellular matrix architecture, and tailoring of mechanical properties are just a few of the desirable properties that are imparted by using nanofibrous scaffolds, all of which are crucial considerations when building scaffolds for a certain organ system [20, 22].

Due to the ability of nerves to conduct electrical signals, there has been significant interest in using synthetic conductive polymers to create nerve guidance conduits (NGCs). Carbon nanotubes (CNTs) have been effectively utilized as promising devices to enhance neuronal signal transmission, as well as to facilitate dendritic elongation and cell adhesion. This distinguishes CNTs among other conductive synthetic materials like polypyrrole (PPy) and polyaniline (PANI) [26, 27].. PANI is infrequently utilized due to conflicting reports of its tendency to induce an exaggerated immune response or persistent inflammation [27]. It has weak mechanical characteristics, and during the early stages of neuron regeneration, this material exhibits reduced conductivity in acidic conditions [28]. PPy is characterized as a hydrophobic substance and Tissue engineering scaffolds made with PPy have limited processability when utilizing conventional methods because the conductive polymer is insoluble, infusible, and brittle [29]. Multiwall carbon nanotubes (MWCNTs) are frequently used in tissue engineering applications. Due to their unique characteristics, including high strength, flexibility, electro-conductivity, and extra functions with different molecules, MWCNTs have shown considerable potential for use in neural system research. Numerous studies have shown that MWCNTs are effective electrical conductors in scaffolds that may alter the conductivity and spatial organization of neurons to affect their growth and differentiation [30]. Moreover, high porosity is a fundamental factor for tissue integration as the natural ECM is extremely porous. By considering this point, MWCNTs are suitable candidates for scaffold production, in low concentrations due to the presence of a high degree of porosity in their structure and being safe or non-toxic for cells [31].

Applying stem cells for peripheral nerve regeneration is one of the most significant research strategies. Neural cells can be differentiated from different sources of stem cells in several circumstances. Endometrial stem cells (EnSCs) have a special potential as therapeutic agents in neural tissue engineering since they are simple to isolate with no additional morbidity, grow quickly without posing serious ethical or technical issues, provide a greater overall clonogenicity, and have the capacity to differentiate into neural cells [32–35]. Several differentiation protocols have been established to steer hEnSCs toward certain neural cells. These protocols take into consideration the various interactions that occur between hormones, growth factors, and other components in the neural system [36]. Mesenchymal stem cells have the ability to transform into neural cells when they are subjected to certain chemical agents (such as growth factors) or mechanical stimuli [37].

Comprehensive knowledge of the variables that initiate and regulate the process of differentiation is essential for the effective implementation of stem cell therapy. When it comes to MSC proliferation and differentiation in vitro, culture conditions are crucial [37, 38].

According to research, better suturability, durability, and mechanical integrity for supporting nerve regeneration can be provided by the bilayer architecture [39]. So, in this study for use in sciatic regeneration, we created a novel bilayer PLA/PU/MWCNT nerve guidance conduit using an inner layer of PU/MWCNT nanofiber mat and an exterior layer of PLA fibrous mat to facilitate the delivery, enhancement of hEnSC engraftment and migration into the damaged side, organization of neuronal networks, and enhancement of neural regeneration. The NGC was made by hand-rolling two layers of electrospun mats. We believe that this effort introduces a new model in sciatic nerve regeneration.

## Methods

### Chemicals and materials

PLA with an average molecular weight (Mw) of 500 kDa (F45989881, Germany) and PU were purchased from Sigma-Aldrich (81,367, Germany). Carboxyl group-Functionalized MWCNTs (MWCNT-COOH) (> 95%, OD: 20-30 nm) were obtained from US NANO (US4302) The Dulbecco’s Modified Eagle’s Medium (DMEM/F12) was purchased from Invitrogen (USA). Fetal bovine serum (FBS, 10270-106), penicillin/streptomycin (15070), and trypsin-EDTA (25300-054) were obtained from Gibco (USA). Chloroform and methanol were obtained from Merck (Germany) company. Phosphate-buffered saline (PBS, P4417), 3-(4,5-dimethylthiazol-2-yl)-2,5-diphenyl tetrazolium bromide (MTT, M2128), 4′,6-diamidino-2-phenylindole (DAPI, D8417), M199 solution were all provided by Sigma-Aldrich (Germany).

### Spinning/spray solution preparation

In order to prepare the scaffold for nerve regeneration, the solution of 10% (w/v) PLA, 8% (w/w) PU, and 10% (w/v) MWCNT should be prepared. For making 10% (w/v) PLA solution, 0.25 g PLA was dissolved in 2.5 ml of chloroform/methanol (75/25) solvent. To reach a homogenous solution of PU, the polymer was dissolved in a chloroform/methanol (70/30) solvent system at 38 °C for 1 hour on the magnetic stirrer. The 10% (w/v) MWCNT–COOH suspension was prepared through dispersion in absolute ethanol (as the solvent) using bath sonication for 3 hours.

### Nanofibers fabrication

#### PU fiber fabrication

**Fig. 1:**
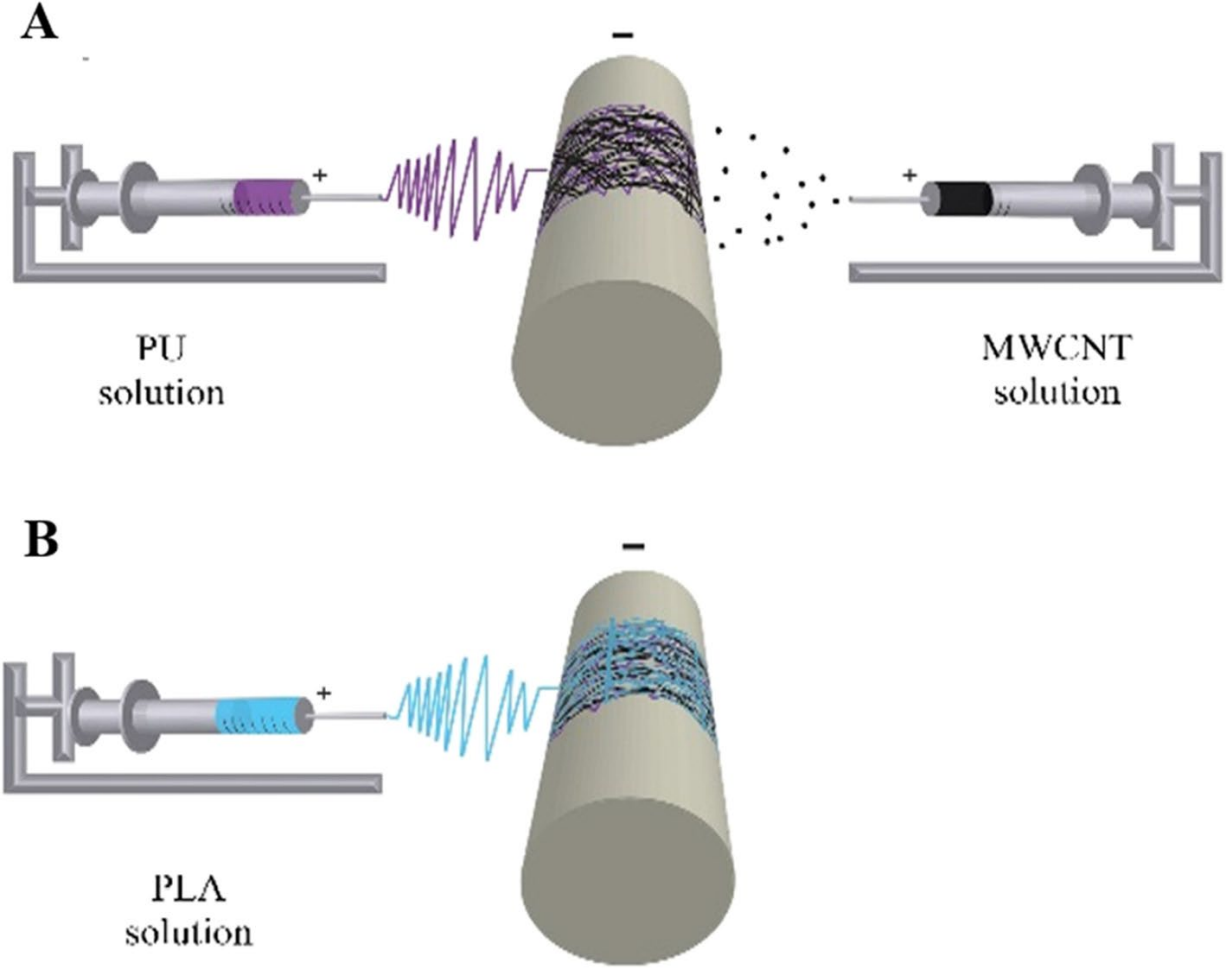
Schematic diagram of (**a**) dual and (**b**) single electrospinning system for fabrication of PU/MWCNT and PLA fibers

A single electrospinning nozzle was used for preparing PU nanofiber film (Fig. 1). The PU solution was loaded into a 5 ml syringe equipped with an 18-gauge needle that was connected to a high-voltage source. On a sheet of aluminum foil, the extruded solution was gathered while it was at room temperature. To produce the aligned nanofibers, the drum speed was established at 1500 rpm/min. The voltage, spinning rate, and collecting distance between collector and needle were fixed as + 14 kV, 0.5 ml/h, and 16 cm, respectively for PU solution injection.

#### Fabrication of PU/MWCNTs fibers

A dual pump electrospinning machine (Side by Side Electroris, FNM, Tehran, Iran) was used to conduct the electrospinning procedure. This system has two scanning systems, two distance adjusters, two high-voltage power supplies, two syringe pumps on either side of the spinning collector drum, and an electrically grounded rotating drum (Fig. 1a). Actually, parallel PU electrospinning and CNT electrospraying were used to fabricate the PU/MWCNTs scaffolds. The prepared solutions were inserted into the 5 ml syringes equipped with an 18-gauge needle with a 1 mm inner diameter. Next, the syringe needles were individually attached to two positive high-voltage power supplies. During the creation of the mesh, the electrospun fibers were gathered on the grounded collecting drum that was covered with aluminum foil. In order to electrospinning the PU solution, a positive voltage of 14 kV, a flow rate of 0.5 ml/h, and a distance of 16 cm between the needle tip and collector were considered. For producing the aligned nanofibers, the drum speed was considered 1500 rpm/min. Furthermore, parameters for electrospraying the CNT solution, were 1 ml/h of flow rate, 8 cm of distance, and 10 kV of applied voltage.

#### PLA fiber production

In order to electrospun the PLA, a 5 ml syringe was filled with PLA solution equipped with an 18-gauge needle that connected to a positive voltage of 16 kV and used a flow rate of 1.0 ml/h.

A distance of 16 cm between the needle tip and the collector was considered. For producing the aligned nanofibers, the drum speed was considered 1500 rpm/min (Fig. 1b).

### Preparation of PLA/PU/MWCNT conduits

In the fabrication of PLA/PU/MWCNT conduits the PLA and PU/MWCNT nanofibrous mats were unwrapped from aluminum foil and rolled up around a 2 mm diameter stainless steel rod. The conduit was constructed with an inner layer of PU/MWCNT fibrous mats and an outside layer of PLA fibrous mats.

### Characterization of fabricated nanofibers

#### Scanning electron microscopy (SEM)

In order to study the morphology and diameter of PLA, PU, and PU/MWCNT nanofibers, a Digital Vacuum Scanning Electron Microscope (AIS2300C SEI, Korea) was used at the accelerating voltage of 15 kV. For this purpose, the surface of nanofiber yarn was sputter-coated with gold and characterized with a scanning electron microscope. To obtain the average fiber diameter of electrospun nanofibers SEM images of 100 randomly selected fibers (*n* = 5) in each sample were investigated using image visualization software Image J (1.52 V).

#### Four-point probe measurements

The electrical conductivity of the electrospun PU/MWCNT nanofibers (*n* = 3) was measured using a four-point probe method (Signatone SYS-301 with Keithley 196 system DDM multimeter). Both before and after adding MWCNTs to the electrospun PU nanofibers, measurements were taken. This was accomplished by applying a voltage to the square sample and measuring the resulting electrical current. In order to determine electrical resistivity (ρ) and conductivity (σ) the following equations were used:

1 $$\uprho ={\textrm{R}}_{\textrm{sh}}.\textrm{t}$$

2 $$\sigma =1/\uprho$$

Where R_sh_ is the electrical resistance of the mat (Ω/sq), t is the sample thickness (cm), ρ indicates the electrical resistivity (Ω.cm), and σ is the electrical conductivity (in S cm − 1).

#### Mechanical properties measurement

For measuring the mechanical characteristics of three samples of each conduit, a universal testing device (SANTAM, sari, Iran) has been applied. The PLA, PU, PU/MWCNT, and PLA/PU/MWCNT conduits were trimmed to a 5 mm wide by 20 mm long rectangle. The tensile test was performed using a load of 10 N and an extension rate of 1 mm/min. The SD of the relevant values was reported, and the ultimate tensile strength and elongation at break were calculated.

#### Contact angle analysis

For scrutinizing the hydrophobicity of fabricated nanofibers, the water contact angle of fibers was measured at three time points for each sample (PLA, PU, and PU/MWCNT nanofibers), using a static contact angle measuring device (KRUSS, Hamburg, Germany). At room temperature, 5 μl of DI water was dropped onto the surfaces of the fibers that had been attached to the coverslips. Within 10 seconds of placing a drop on each sample, the tangent line at the water droplet’s point of contact with the surface was measured. The information was presented as a mean SD.

#### Degradation rate and PH variation measurement

In order to study in vitro degradation, the PLA, PU, PU/MWCNT, and PLA/PU/MWCNT conduits were submerged in 5 mL of PBS (pH 7.4) at 37 °C. At each time point (weeks 1, 2, 4, 6, and 8) three samples were taken out of PBS, rinsed with distilled water, allowed to air dry, and the weight changes were monitored to determine the degree of degradation. By utilizing the eq. (3) To get the weight-loss data, the degradation was quantified. In this equation, W_0_ and W_1_ represent the conduit’s original and dry weight upon removal from the PBS, respectively. Each conduit’s three weight loss values were averaged.

3 $$Weight\ loss\ \left(\%\right)=\frac{W_0-{W}_1}{W_0}\times 100$$

Furthermore, after each time point (weeks 1, 2, 4, 6, and 8) variation of pH during the degradation of each sample was evaluated with a digital pH meter (ZAG CHEME CO Model PTR79).

#### Swelling ratio of nanofibrous conduits

For evaluating the fiber swelling amounts, under static conditions, PLA, PU, PU/MWCNT, and PLA/PU/MWCNT conduits with specified weights were incubated in PBS solution at 37 °C for 48 hours until the equilibrium swelling state was reached. The enlarged fibers were taken out of the solution at the specified times (*n* = 3), and tissue paper was used to absorb the extra water. The samples were weighed right away using a microbalance. In order to calculate the swelling ratio, eq. (4) has been applied. W_s_ is the weight of the samples after the swelling equilibrium and W_i_ is the initial weight of the samples.

4 $$Swelling\ Ratio\ \left(\%\right)=\frac{W_s-{W}_i}{W_i}\times 100\kern0.5em$$

#### Porosity measurement

The liquid displacement method was applied for conduits’ porosity assessment, via the following equation.

5 $$Porosity=\left(\%\right)\frac{v1-v3}{v2-v3}\times 100$$

After soaking the conduits in deionized water, the liquid’s volume increases from V1 (initial volume) to V2. When the conduit is removed, the liquid’s volume decreases to V3. For each conduit, we averaged three different porosity percentages.

#### Fourier transform infrared spectroscopy (FTIR)

For evaluating the chemical structure, functional groups, and possible interaction between each component of the composite scaffolds, the FTIR technique (Cary630-Agilent, USA) with a scan range of 500–4000 cm − 1 and a resolution of 4 cm − 1 has been used.

### Cell culture studies

#### Culture of human endometrial stem cells

hEnSCs were provided by the Iranian Biological Resource Center. Cells were cultured in DMEM/F12 with 10% FBS and 100 units/mL of penicillin/streptomycin in a CO_2_ incubator at 37 °C with 5% CO2. The medium was changed every 3 days. The cells were at 80% confluency. For the experiments, cells at passage 3 were utilized.

#### Cell seeding on the scaffolds

Fibrous mats were cut to the size of each well in the 48 and 96-well culture plates and placed on them. After that, the nanofibrous scaffolds were sterilized under UV light for 30 min, were washed with PBS (pH 7.4) containing 1% penicillin/streptomycin and 1% amphotericin B, and then were soaked in DMEM/F12 medium containing 10% FBS and 1% penicillin/streptomycin overnight at 37 °C. EnSCs (6 × 10^4^ and 10^4^ cells, respectively) exactly were cultured on the surface of nanofibrous scaffolds in each well of the 48-well and 96-well culture plates. After 2 hours, samples were supplemented with DMEM/F12 containing 10% FBS and incubated at 37 °C and 5% CO2.

#### Cell attachment and morphology

To explore the potential for the development of a connection between the cell and constructed polymeric scaffolds, an analysis of cell adhesion on polymeric scaffolds was carried out using an SEM (AIS2300C SEI, Korea) after 2 days of culture [40, 41]. The medium used to grow the cells was removed, and PBS was used to wash the cells. In order to dehydrate the cells on polymeric scaffolds, samples were first fixed using Karnovsky’s Fixative (2% (w/v) paraformaldehyde and 2.5% (w/v) glutaraldehyde) for 40 minutes at room temperature. Gradual concentrations increasing of ethanol solutions were then applied from 30 to 100% for 3 min for each condition. In order to examine the polymeric scaffolds, an SEM coating of gold was applied. Three samples were used for each group.

#### Cell viability and proliferation

To assess cell proliferation and viability, in each well of 96-well plates (with and without scaffolds), 10^4^ cells were seeded and incubated at 37 °C. To evaluate the viability of cells on various polymeric scaffolds and the effect of scaffolds on cells, the MTT assay was carried out after 1, 3, and 5 days of cell seeding in the cell culture plate, PLA, PU, PU/MWCNT, and PLA/PU/MWCNT scaffolds (*n* = 3). Each well received 200 μl of 0.5 mg/ml MTT solution after the medium removal and PBS washing. After 4 hours of incubation, the MTT solution was eliminated and 100 μl of DMSO was added, gently mixed, and agitated for 10 minutes in a dark environment to dissolve any formazan crystals that had formed. For assessing the samples’ absorbance, an ELISA reader (Expert 96, Asys Hitch, Ec Austria) was used at 570 nm.

DAPI cell staining was utilized to evaluate cell adhesion 1, 3, and 5 days after cell implantation on scaffolds by staining the nuclei of hEnSCs. DAPI staining was accomplished by removing the culture medium, washing the polymeric scaffolds with PBS, fixing them with 4% paraformaldehyde at room temperature for 30 minutes, rinsing them once more with PBS, and staining them with DAPI (10 g/ml) for 5 min in the dark. The DAPI solution was replaced with PBS, and a fluorescence microscope (H600L, Optika, Italy) was used to check for the presence of cell nuclei.

### Statistical analysis

Data were analyzed using SPSS software. One-way analysis of variance and Dunnett’s two-tailed t-test (DUNNETT) were employed to evaluate the statistical significance of differences between the control and all experimental groups. All data have been presented as mean standard deviation (SD). Differences were considered statistically significant at *p < 0.05, **p < 0.01, and ***p < 0.001.

## Results

### Characterization of scaffolds

#### Scanning electron microscopy (SEM)

**Fig. 2:**
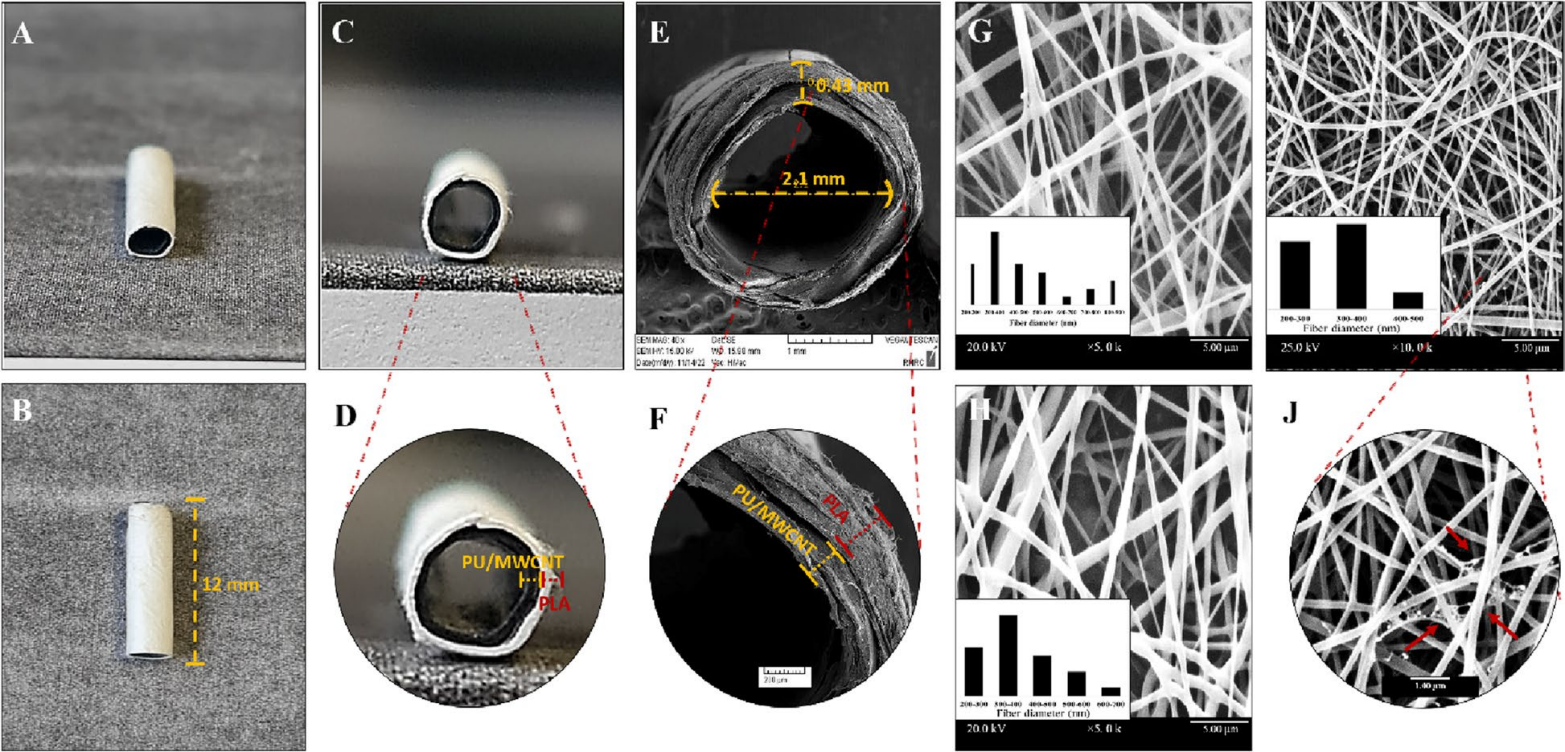
Macrographs of nanofibrous nerve construct. **a**-**d** Bilayer nanofibre nerve conduit; (**e**, **f**) scanning electron micrographs (SEMs) of bilayer nanofibrous nerve conduit at different magnifications; (**g**-**j**) SEM images of the electrospun nanofibers at different magnifications demonstrating porosity and homogeneous fibrous structures in (**g**) PLA, (**h**) PU, and (**i**, **j**) PU/MWCNT nanofibrous mats. Multiwalled carbon nanotubes (MWCNTs) embedded in nanofibres are denoted by Red arrows

According to the SEM findings (Fig. 2), the average diameters of PLA, PU, and PU/MWCNT were 400.57, 380.18 nm, and 311.47 nm, respectively, and polydispersity indexes were 0.22 and 0.28. Despite their alignment and diameter, the resulting fibers were continuous, beadless, and had a smooth uniform surface. However, it can be observed that there is a reasonable decrease in PU fiber diameters due to the addition of MWCNTs. Moreover, the morphology of PLA and PU nanofibers with and without MWCNTs is represented in Fig. 2.

#### Electrical measurements

Using a four-point probe, the resistance and electrical conductivity of the pure PU and PU/MWCNT nanofibrous mats were measured and computed in accordance with eq. (1 and 2) as depicted in Table 1. Because MWCNTs have a high electrical conductivity, they can be added to pure nanofibers to increase conductivity, which is a crucial quality of electroconductive scaffolds. The data showed that electrospraying of MWCNT significantly improved the electrical conductivity of PU nanofibers (*p* < 0.001).

**Table 1:** The resistance and electrical conductivity of the PU and PU/MWCNT nanofibrous sheets

Polymer	Electrical resistivity (ρ) Ω.cm	Electrical conductivity (σ) S.cm^**−1**^
PU PU/MWCNT	5.8 × 10^9^ 7.8	1.7 × 10^−10^ 1.2 × 10^−1^

#### Tensile test analysis

**Fig. 3:**
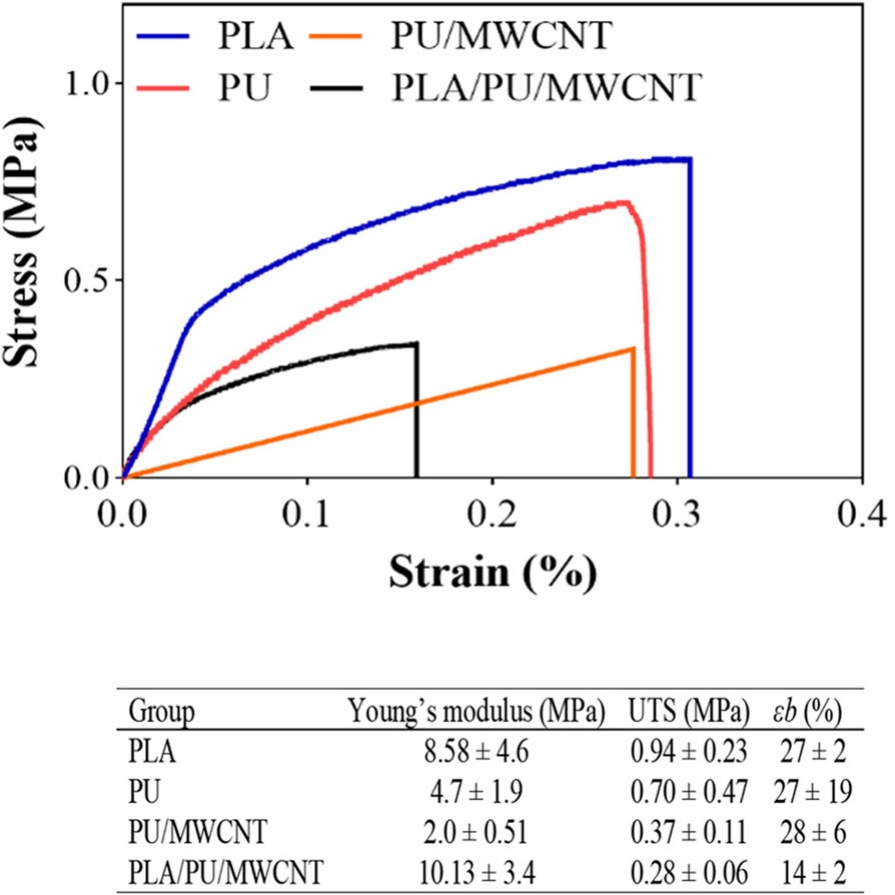
The mechanical attributes of the PLA, PU, PU/MWCNT, and PLA/PU/MWCNT conduits. Tensile properties measurement under 10 N tensile load and an extension rate of 1 mm/min at 37 °C. Compared to PLA/PU/MWCNT scaffolds, PLA (P < 0.05), PU (*P* > 0.05), and PU/MWCNT (P > 0.05) nanofibrous conduits had a lower tensile strength. UTS: ultimate tensile strength, Ɛb: elongation a break. Data are presented as mean SD, *n* = 3

The results of the tensile strength testing demonstrated that compared to PLA/PU/MWCNT scaffolds, conduits made just of PLA (*P* < 0.05), PU (*P* > 0.05), or PU/MWCNT (P > 0.05) had a substantially lower tensile strength (Fig. 3).

In comparison to mats made of composite nanofibers, the PU/MWCNT mats displayed weak mechanical properties (E = 2 MPa). PLA was incorporated into PU/MWCNT fibers to enhance their mechanical qualities (P < 0.05).

#### Water contact angle analysis

**Fig. 4:**
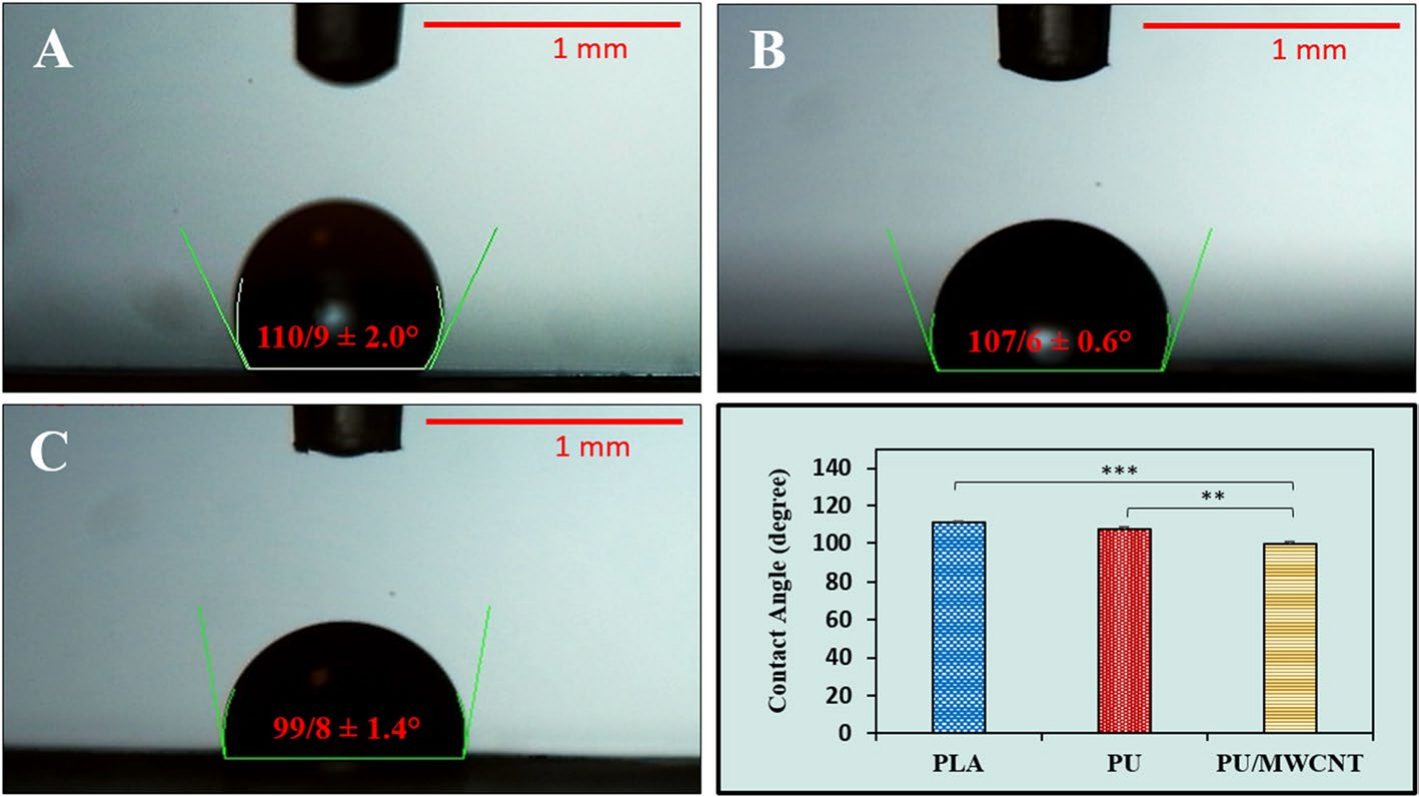
Average water contact angle of the electrospun (**A**) PLA, (**B**) PU, and (**C**) PU/MWCNT nanofibrous mats. The addition of MWCNTs enhanced the hydrophilicity of PU nanofibers. Data are reported as mean SD, *n* = 3 (***p < 0.001, **p < 0.01)

The initial cell adhesion, proliferation, and migration can be affected by the surface behavior of fibrous scaffolds [12]. This characteristic of the scaffold was measured using a contact angle by applying deionized water. Figure 4 depicts the typical water contact angle of PLA, PU, and PU/MWCNT nanofibrous mats. As it is depicted, PU/MWCNT nanofibrous mats had average water contact angles that were lower than those of PU and PLA nanofibrous mats (99.8 ± 1.4° vs. 107.6 ± 0.6° and 110.9 ± 2.0°, respectively). Measurement of contact angles revealed a considerable difference between the fabricated scaffolds. According to the findings, the addition of functionalized MWCNTs to PU nanofibrous mats reduced their surface hydrophobicity significantly.

#### In vitro degradation rate and pH variation

**Fig. 5:**
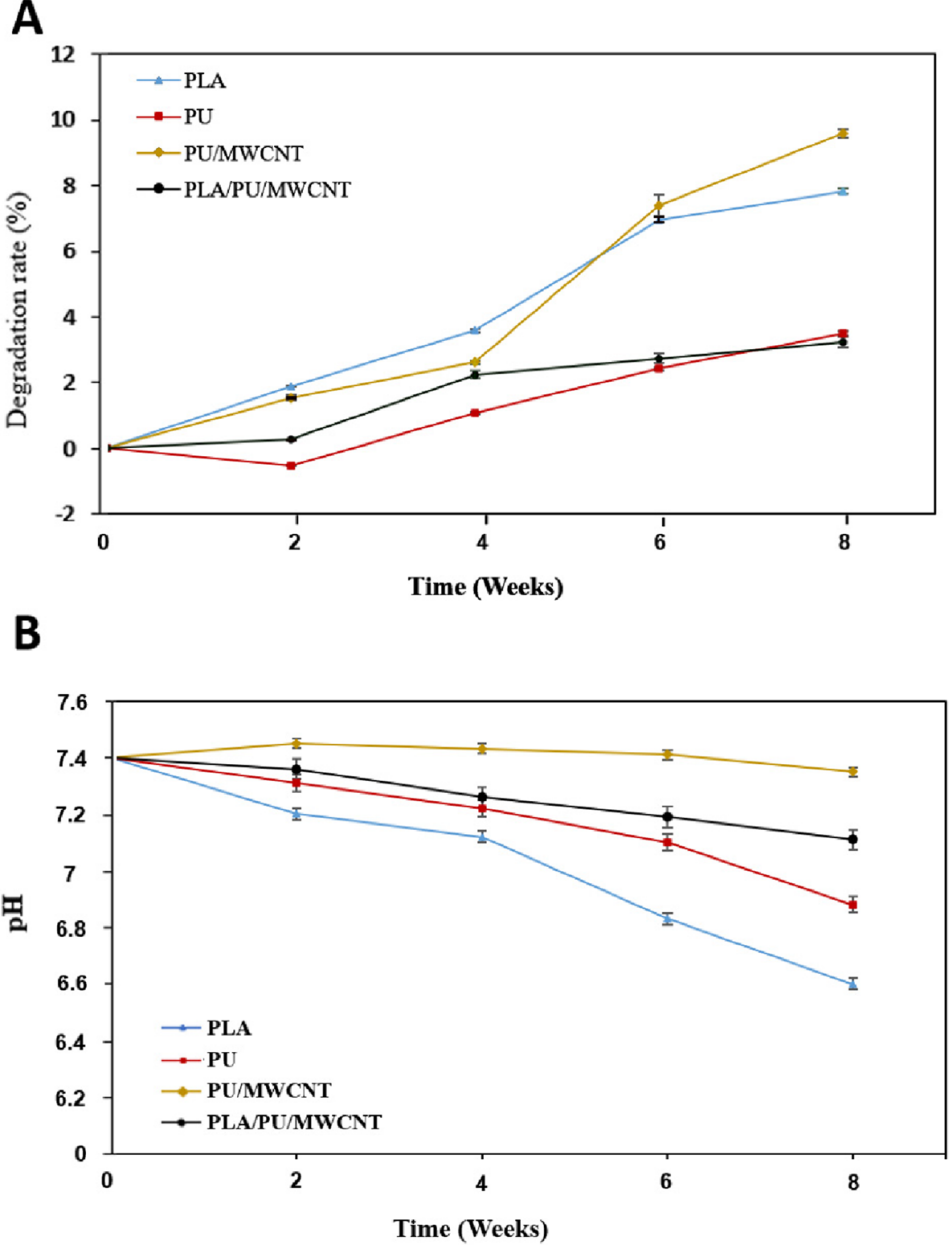
The rate of degradation (**a**) and pH variation (**b**) of PLA, PU, PU/MWCNT, and PLA/PU/MWCNT conduits during 8 weeks of sucking in PBS (pH 7.4) at 37 °C. **a** In all groups, the degradation rates show a significant change during eight weeks (*p* < 0.05). **b** In all groups, we can see a little decrease in pH value over eight weeks but without statistical significance (*P* > 0.05). The data are shown as mean SD, (*n* = 3)

Monitoring of the in vitro degradation process involved the analysis of pH change and mass loss. Figure 5 shows the changes in weight and pH over the 8 weeks.

Figure 5a shows the conduits in PBS average weight-loss percentages after each time point. The degradation rate of all groups shows a significant change over 8 weeks (*p* < 0.05). After 8 weeks, the PLA conduit’s weight loss percentage showed that it had lost relatively little weight. After 8 weeks, the weight-loss percentage of the PU conduit showed that there had been a slight weight loss. The addition of MWCNTs greatly accelerated the degradation of the PU conduit, raising it to 2.61% (after 4 weeks) and 9.58% (after 8 weeks) as a result of the increase of material hydrophilicity and increased contact with water. The weight of the mixture of PLAs and PU/MWCNT conduit was reduced after 8 weeks.

For the following scaffolds; PLA, PU, PU/MWCNT, and PLA/PU/MWCNT, Fig. 5b depicts the pH variation, which is maintained nearly without alterations until week 4, after which it declined fast, reaching values at the end of week 8 of 6.6, 6.88, 7.35, and 7.11, respectively (*P* > 0.05). The samples without nanotubes showed a greater decline in this quantity.

#### Swelling ratio of conduits

**Fig. 6:**
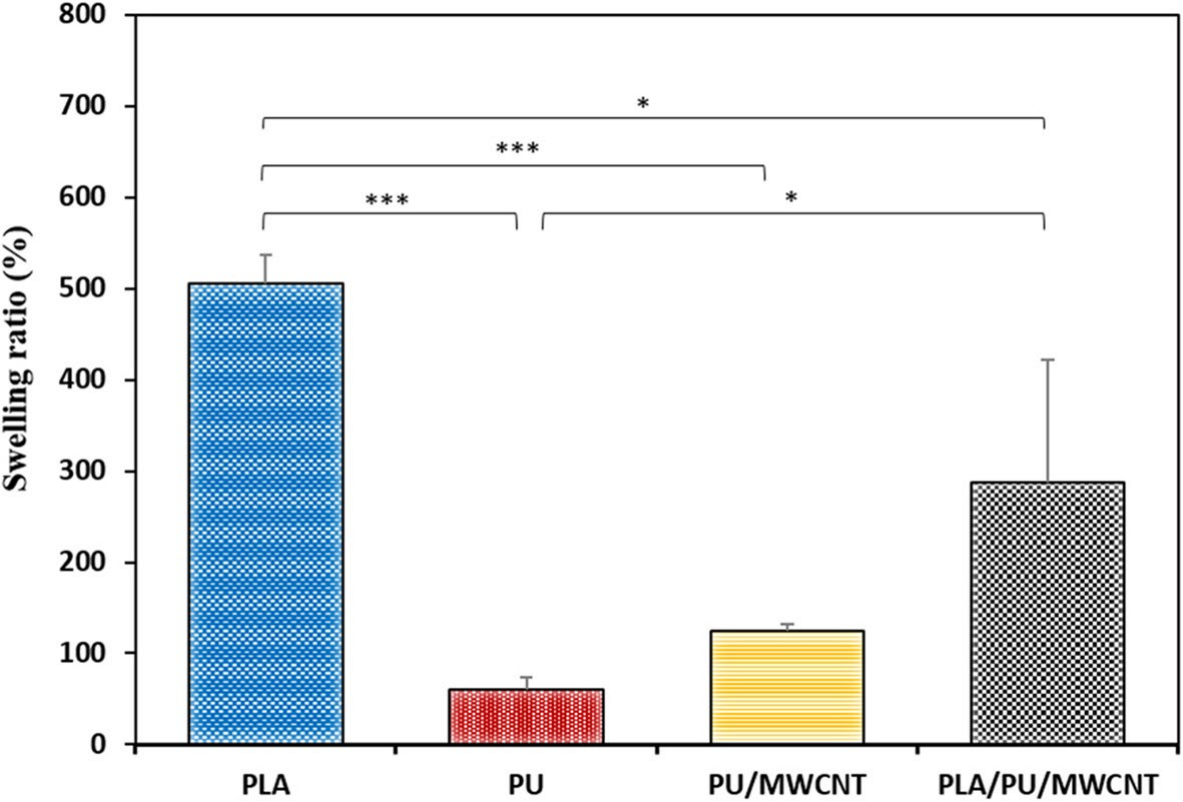
Swelling ratios of the PLA, PU, PU/MWCNT, and PLA/PU/MWCNT nanofibrous scaffolds after 48 hours of incubation at 37 °C with PBS (pH 7.4). Data are presented as mean SD, *n* = 3 (***p < 0.001, **p < 0.01, *p < 0.05)

Polymer interactions, surface behavior, and crosslinking density can all affect the degree of scaffold swelling [42–45]. Figure 6 displays the percentages of water absorption of polymeric conduits over 2 days of being sucked into PBS. According to the Figure, PLA and PU nanofibrous conduits indicated higher and lower water absorption, in comparison with the other nanofibers. As can be seen in the Figure, PU/MWCNT scaffolds showed higher swelling compared to PU conduits, even though the difference was not significant. The amount of water absorbed by PLA/PU/MWCNT nanofibrous scaffolds was greater than that of groups with PU and lower than that of PLA scaffolds.

#### Porosity measurement

**Fig. 7:**
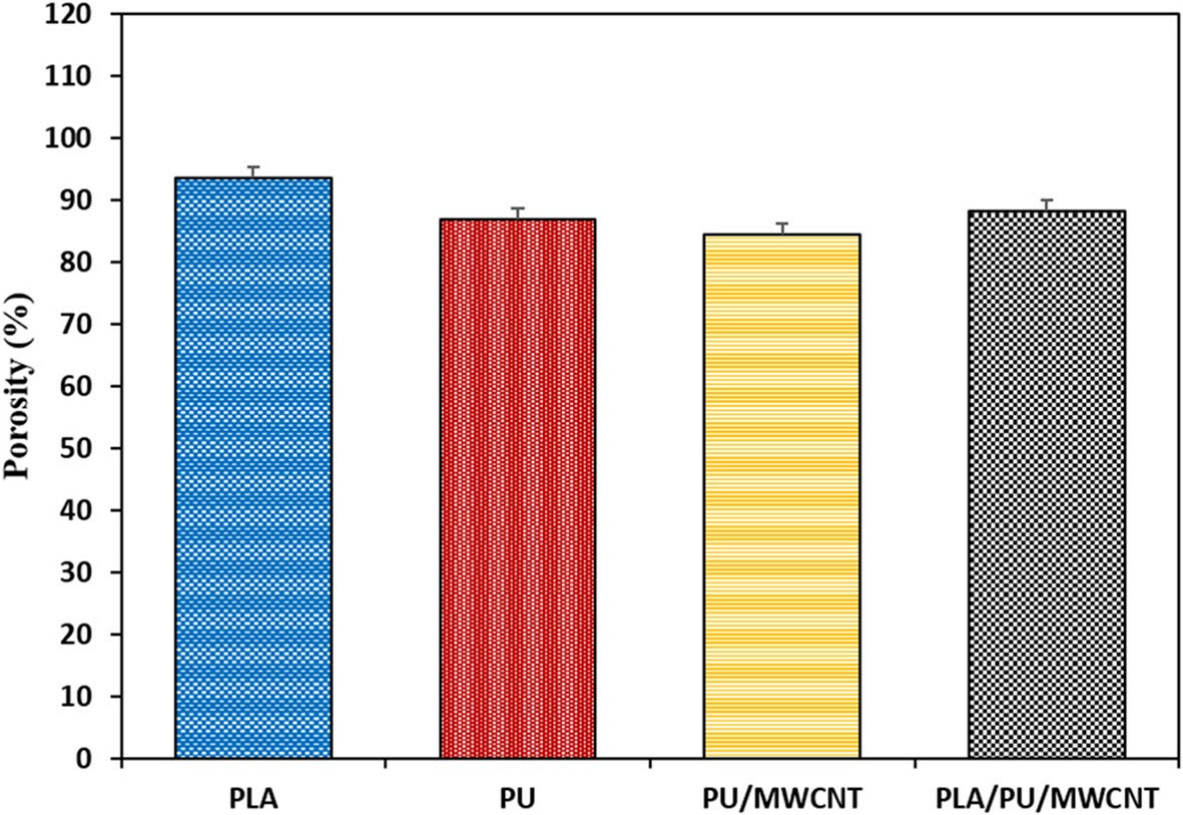
The porosity of the PLA, PU, PU/MWCNT, and PLA/PU/MWCNT nanofibrous scaffolds. The PLA conduit to the PU, PU/MWCNT, and PLA/PU/MWCNT conduits, it was shown to have more porosity, but without statistical significance (P > 0.05). Data are presented as mean SD, n = 3

The porosity of the prepared conduits is sufficiently higher (> 80%) in order to use them for tissue engineering [12]. The PLA conduit revealed higher porosity (93.5%) compared to the PU, PU/MWCNT, and PLA/PU/MWCNTs (Fig. 7). However the differences are not significant (*P* > 0.05).

#### FTIR analysis

**Fig. 8:**
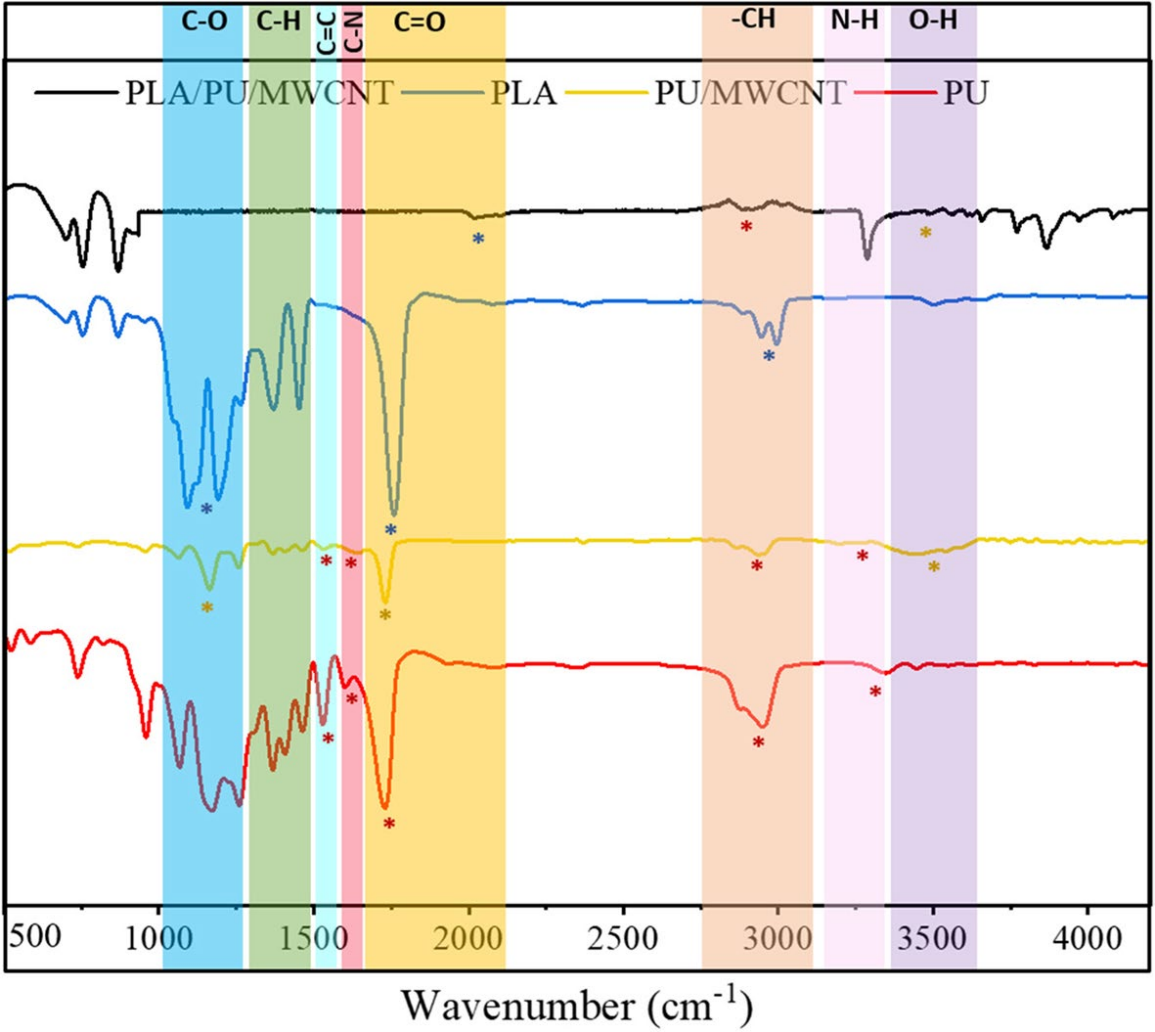
The FTIR-ATR spectroscopy. Chemical structure of PLA, PU, PU/MWCNT, and PLA/PU/MWCNT nanofibers. Remarkable functional groups in each sample are denoted by stars

FTIR characterization of PLA, PU, PU/MWCNTs, and the PLAPU/MWCNT composite was also conducted and shown in Fig. 8. FTIR results of PU displayed prominent distinctive peaks of C=O, C-H, N-H, C-N, and C=C. The PU/MWCNTs composite sample revealed the parent type of PU in the composite as well due to the presence of all results’ peaks. The O-H peaks in the PU/MWCNTs samples were not present in the PU samples. FTIR research confirmed that MWCNTs can reinforce the PU/MWCNT composite material. The results demonstrated that the samples were prepared correctly.

### Cell study

#### Cell adherence to scaffolds

**Fig. 9:**
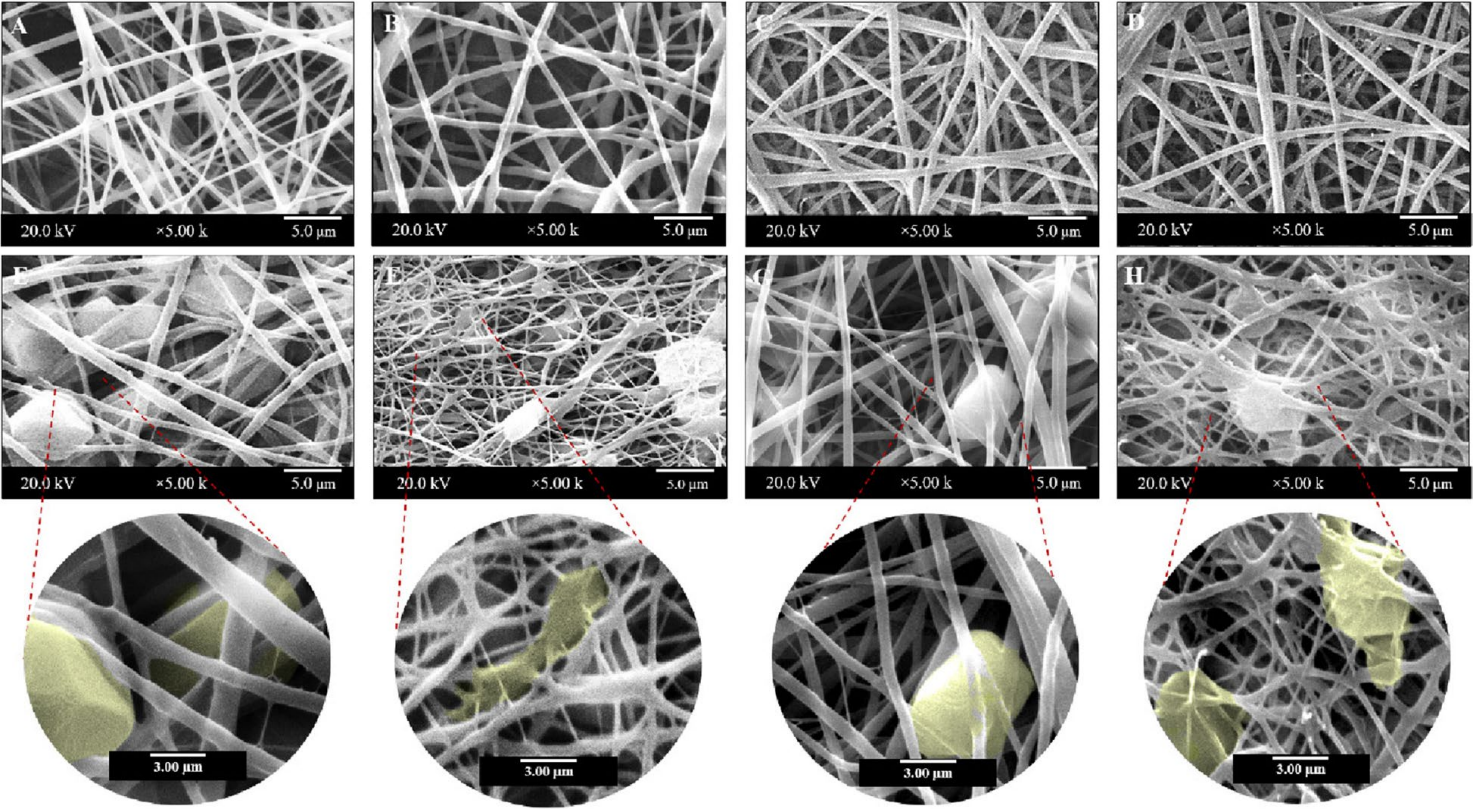
SEM images of hEnSCs cultured on PLA, PU, PU/MWCNT, and PLA/PU/MWCNT nanofibrous scaffolds after 48 hours. (A–D) Scaffolds without cells; (E–H) scaffolds with cells (pseudo-colored) at different magnifications

SEM was used to examine cell morphology and interactions of the hEnSCs on the scaffolds after two days (Fig. 9). SEM showed that hEnSCs adhered to and spread across all of the scaffolds.

These photos show interactions between scaffolds and cells as well as the adhesion of cultured cells to the scaffolds. A handy approach to promote the supply of enough oxygen and nutrients to the implanted construct and cultured cells can be found in the porous microstructure of scaffolds.

#### Cell proliferation and viability

**Fig. 10:**
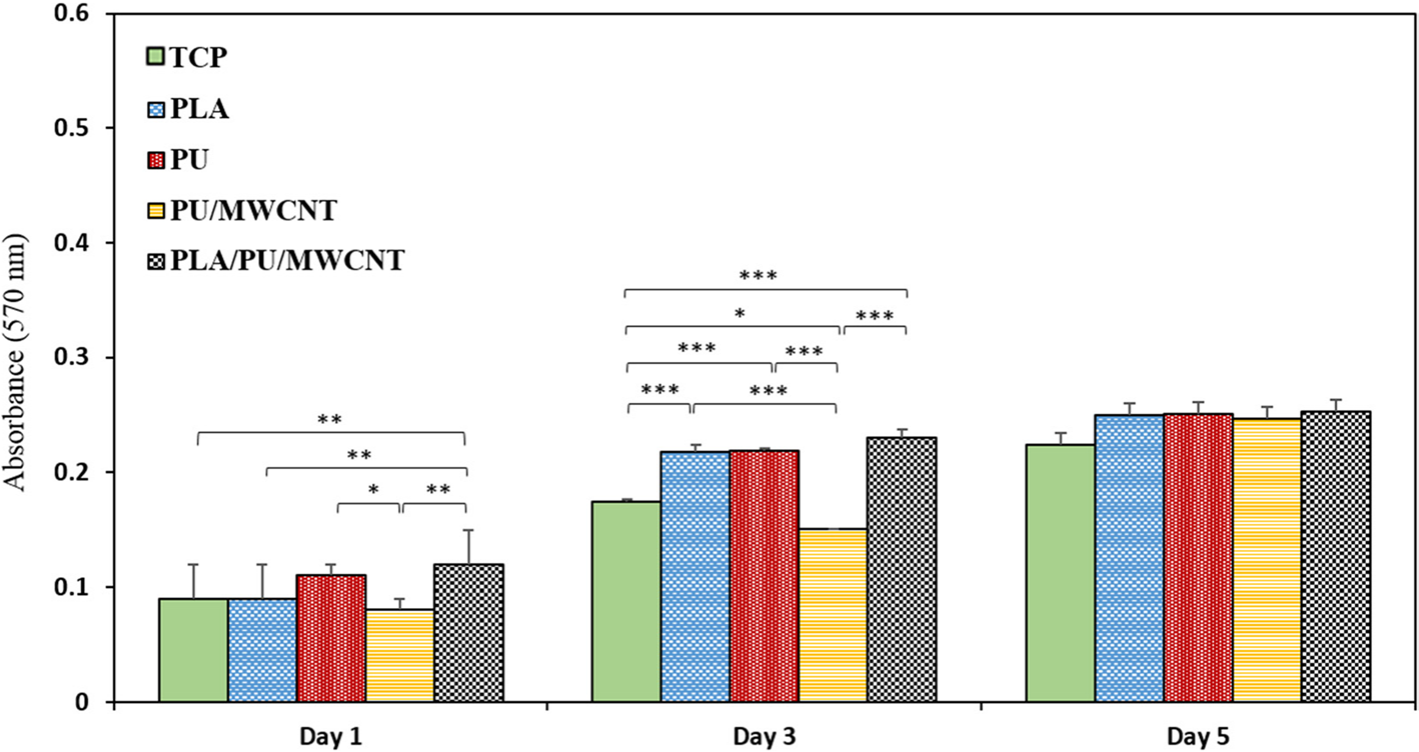
MTT assay. The viability of hEnSCs cells seeded on tissue culture plate (TCP) and different nanofibrous scaffolds after 1, 3, and 5 days. Data are presented as mean SD, *n* = 3 (***p < 0.001, **p < 0.01, *p < 0.05)

The persistence of cells following attachment and growth onto the nanocomposite mats was quantitatively assessed using the MTT test. The amount of purple formazan crystals generated in this assay is related to the quantity of live cells. The optimum solvent for dissolving the formazan product and producing a purple color is DMSO. Data from the MTT assay are shown in Fig. 10 on days 1, 3, and 5. At all three time points, good cytocompatibility was seen in all groups. On the other hand, compared to the cell culture plate, the PLA/PU/MWCNT nanofibrous scaffolds demonstrated a higher level of cytocompatibility, and these differences were significant on the first and third days.

**Fig. 11:**
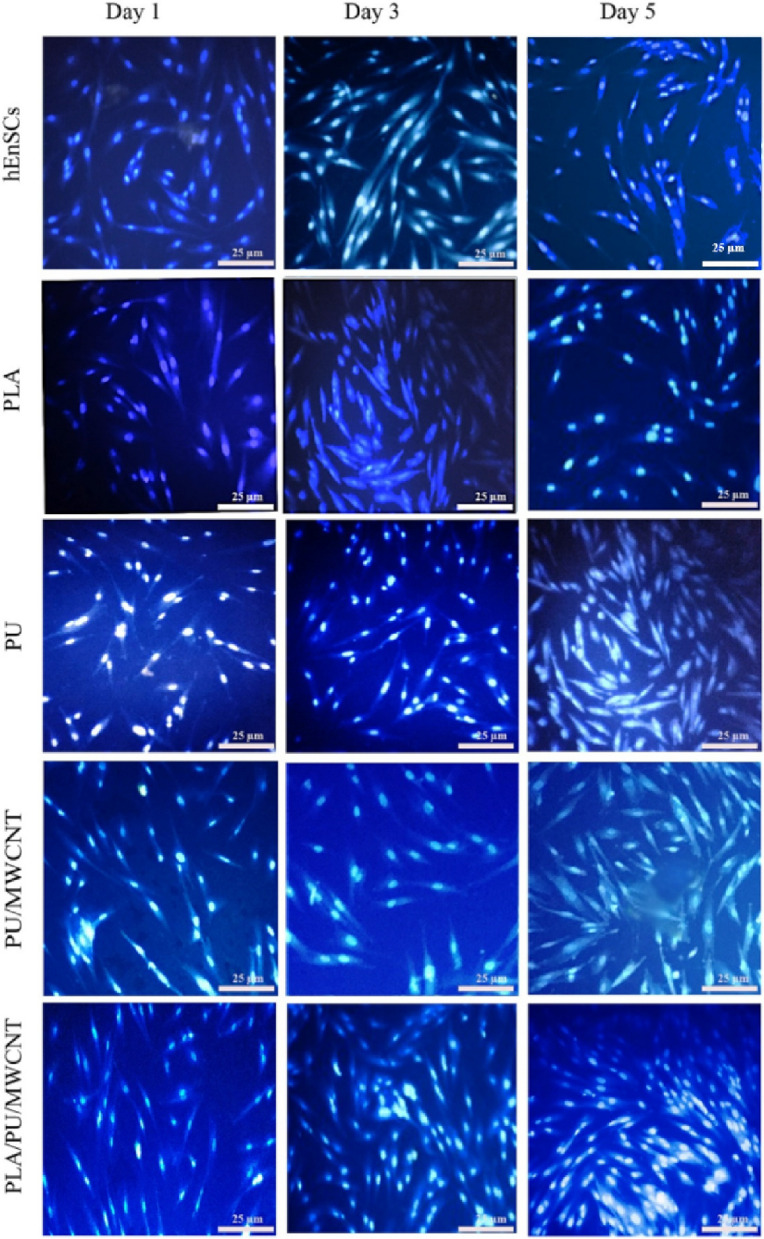
Fluorescent images of hEnSCs stained with DAPI seeded on tissue culture plate (TCP) and scaffolds after 1, 3, and 5 days. The stained cell’s nuclei were blue under fluorescence microscopy

Fluorescent pictures of the cells stained with DAPI cultured in tissue culture plate (TCP), PLA, PU, PU/MWCNT, and PLA/PU/MWCNT nanofibrous scaffolds were taken and shown in Fig. 11. In the fluorescence microscope, the nuclei could be seen in blue. In comparison to PLA, and PU samples, more cells had attached to the PU/MWCNT and PLA/PU/MWCNT samples after 24 hours of cell seeding.

## Discussions

Allografts and autografts are options for treating traumatic nerve injuries with wide gaps, but they have illustrated some drawbacks. Engineering a fully degradable NGC with acceptable mechanical, physical, and biological qualities will satisfy the requirements for creating the next generation of biomaterials for sciatic nerve regeneration applications [46]. Recently electrospun nanofibrous conduits have been used for nerve tissue engineering [47]. The fibrous structure closely resembles the natural environment which cells grow and provides appropriate physical cues for manipulating cellular functions. Previous studies have not reported the procedure, design, and construction of bilayer PLA/PU/MWCNT scaffolds for PNS tissue engineering. Bilayer PLA/PU/MWCNT nanofibrous conduits are made by electrospinning as a favorable nerve conduit material to support nerve regeneration. In comparison with conduits obtained by solvent casting, electrospinning gives rise to entirely interconnected porous structures by collecting fibers on cylindrical rotating substrates [48]. The diameter of the scaffold’s fibers is a crucial factor in regulating cell adhesion and differentiation. In comparison to PU fibers treated with CNTs, the average diameter of pure PU fibers is marginally larger. Since the CNT boosted the PU’s electrical conductivity, the electrical field’s advantageous jet splitting and lower fiber widths were both produced [41, 49–51]. The electric properties of MWCNTs have been shown to promote neural cell functions like adhesion, development, and differentiation through effective signal transduction and efficient electron transfer [25, 41, 52].

Conduits’ mechanical strength is a crucial characteristic, since weak conduits may collapse after implantation or rupture during suturing. Our results showed that using polymeric conduits made of the combination of PLAs, PU, and MWCNTs can create an ideal milieu for the regeneration of sciatic tissue. Nanofibers made of a polymer and CNT are fully dependent on CNT concentrations and dispersion quality for their mechanical characteristics [53]. The natural alignment of the nanofibers is supported by the homogeneous dispersion of a relatively low CNT concentration in the polymer matrix, increasing material stiffness [54, 55]. On the other hand, a relatively high CNT concentration causes more heterogeneous dispersion and CNT bundle formation, which is followed by an increase in the nanoroughness of the nanofiber surfaces [56]. In this study, we showed that the incorporation of carbon nanotubes into nanofibers can change their topographical and mechanical characteristics.

In order for biomaterials to interact with cells and be biocompatible, their surface’s hydrophilicity is crucial [57]. The functionalized MWCNTs in the nanofibrous scaffold provided additional responding sites that allowed the droplet to expand. As a result, PU/MWCNT scaffolds’ contact angles were less than those of PU nanofibrous scaffolds. The electrical conductivity of nanocomposites can be necessary for cell signaling or as an electrical signal pathway in some applications such as nerve tissue scaffolds. The electric properties of MWCNTs hone electron transfer, can certainly be of help in effective signal transfer, and play an essential role in enhancing neural cell adhesion, proliferation, growth, and differentiation [25, 52]. In this study, we showed that the electrical conductivity of the constructs was increased with the addition of carbon nanotubes as an engineering important component in neural tissue engineering.

The samples in this investigation that contained MWCNTs showed pH values that were closest to the initial PBS level (pH = 7.4), with pH values decreasing as the nanotube concentration level increased. MWCNTs have a pH-modulating effect, which may help to inhibit the normal inflammatory processes brought on by an acidic pH [58].

Conduits’ mechanical properties as scaffolds for neural tissue engineering are affected by their degradation and swelling ratio. When regenerating the nervous system, scaffolds should have a low swelling ratio and slow degradation rate. The degradation period should be lengthy enough for axons to grow into the injury site during neural cell growth and regeneration. Some scaffolds can swell and lose their mechanical strength before neural regrowth if they break down too quickly. On the other hand, a scaffold that breaks down too slowly may not be able to keep up with the rate of neural growth and regeneration, which can cause a foreign body response [41, 59, 60].

The first 10-12 weeks after injury are crucial for regenerated axons to develop through the distal nerve stump for maximum functional recovery of transected peripheral nerves [61, 62]. A somewhat “undisturbed” environment is needed throughout this time, and nerve conduit degradation is favored subsequently [27]. Results showed that over an 8-week period, PLA and PU/MWCNT scaffolds degraded at a faster rate than PU and PLA/PU/MWCNT scaffolds. These findings corroborated those of a prior study showing that the degradation rate of PU/MWCNTs nanofibers was significantly higher than that of pure PU nanofibers [63]. Finally, the degradation of PLA/PU/MWCNT conduits was examined, and we found that the composite experienced a mass loss ratio that was significantly lower than the pure PLA and PU/MWCNT. These features suggest that it could be useful as a tissue engineering scaffold [64]. Hence, the rate at which the electrospun nerve conduits degrade can be precisely adjusted to create a milieu that is relatively “undisturbed” and conducive to promoting peripheral nerve regeneration.

Due to the large specific surface area of electrospun nanofibers, the inclusion of MWCNTs has a positive impact on the amount of water absorption [65]. Moreover, the PLA/PU/MWCNT conduits had a lower water absorption capacity than the PLA conduits and greater than the PU conduits. Thus, our synthesized nanofibrous composites may be better options for neural tissue engineering due to their averaged swelling ratios [59]. The produced conduits’ porosity is sufficiently greater (> 80%), as shown in the results, for them to be used for tissue engineering applications [66].

Because of their resemblance to the physical characteristics of a natural extracellular matrix, nanofibrous scaffolds provide the ideal environment for cell adhesion and cell development. For the cell culture study, electrospun fibrous conduits were used because they offer a significant surface area that permits cell adhesion [67]. Here, we show how an electrospun PLA/PU/MWCNT porous nerve conduit can promote successful peripheral nerve regeneration thanks to its large surface area for cell adhesion. Additionally, by improving the surface properties of the MWCNT and giving it appropriate hydrophilicity and protein adsorption, cell adhesion and proliferation might be enhanced.

Results of our MTT assay demonstrated that our conduit had no cytotoxic effects on the viability of the cells after 5 days, which was consistent with earlier research [68]. Others have also claimed that by promoting cell growth, proliferation, and extracellular collagen secretion, electrospun nanofibrous composites of PU/MWCNT could replicate the shape and function of extracellular [69]. Additionally, DAPI staining showed that many hEnSCs were attached to the PU/MWCNT and PLA/PU/CNT scaffolds during the course of 5 days, which supported the results of the MTT. According to the theory, the presence of MWCNTs on the surface of PU might produce nanoscale roughness and thus increase protein absorption, which would enhance cell adhesion, proliferation, and differentiation [70, 71]. Moreover, the addition of MWCNTs to the scaffolds may be successful at transferring electrical impulses, turning the electrically nonconductive nanofibers into conductive scaffolds, and improving their cytocompatibility.

## Conclusions

In the current study, an electro-conductive and biodegradable electrospun/sprayed scaffold was effectively constructed and tested. According to the results of PLA/PU/MWCNT scaffold characterization, generated structures might be promising in biomedical applications, particularly in neural tissue engineering. When MWCNTs were electrosprayed on the developing electrospun nanofibers, it gave scaffolds electro-conductive properties. MWCNTs were used as an electroactive and conductive filler. The produced structures also demonstrated appropriate mechanical and wettability characteristics for neural tissue engineering. Additionally, it was shown that the biocompatibility of the conductive electrospun nanofibrous scaffolds for supporting cell adhesion and proliferation was adequate. The outcomes illustrated the scaffolds’ potential for use in applications for peripheral nerve defects.

## Data Availability

No datasets were generated or analysed during the current study.

## Abbreviations

DAPI: 4′,6-diamidino-2-phenylindole
DMEM: Dulbecco’s Modified Eagle’s Medium
ECM: Extracellular matrix
EnSCs: Endometrial stem cells
FBS: Fetal bovine serum
FTIR: Fourier transform infrared spectroscopy
hEnSCs: Human endometrial stem cells
MTT: 3-(4,5-dimethylthiazol-2-yl)-2,5-diphenyl tetrazolium bromide
Mw: Molecular weight
MWCNT- COOH: Carboxyl group-Functionalized multiwall carbon nanotube
MWCNTs: Multiwall carbon nanotubes
NGCs: Nerve guidance conduits
PANI: Polyaniline
PBS: Phosphate-buffered saline
PCL: Poly (caprolactone)
PGA: Poly (glycolic acid)
PLA: Poly (lactide acid)
PLA/PU/MWCNT: Poly-lactic acid/polyurethane/multiwall carbon nanotube (bilayer nanofibrous nerve conduit)
PLCL: Polylactide-caprolactone
PLGA: Poly (lactic-co-glycolic acid)
PNI: Peripheral nerve injuries
PPy: Polypyrrole
PTFE: Polytetrafluoroethylene
PU: Polyurethane
PU/MWCNT: Polyurethane/multiwall carbon nanotube
SEM: Scanning electron microscopy
TCP: Tissue culture plate
